# Supplementary material for: The interactome of intact mitochondria by cross-linking mass spectrometry provides evidence for coexisting respiratory supercomplexes
Source: Mol Cell Proteomics. 2017 Dec 8;17(2):216–32. doi: 10.1074/mcp.RA117.000470 (PMC5795388; doi:10.1074/mcp.RA117.000470)
Supplement: Supplemental Data [file supp_RA117.000470_133922_0_supp_23978_fzffwf.zip › spectra_annotation/mito_spectra_annotation/228-1-4-1-5-1.pdf]

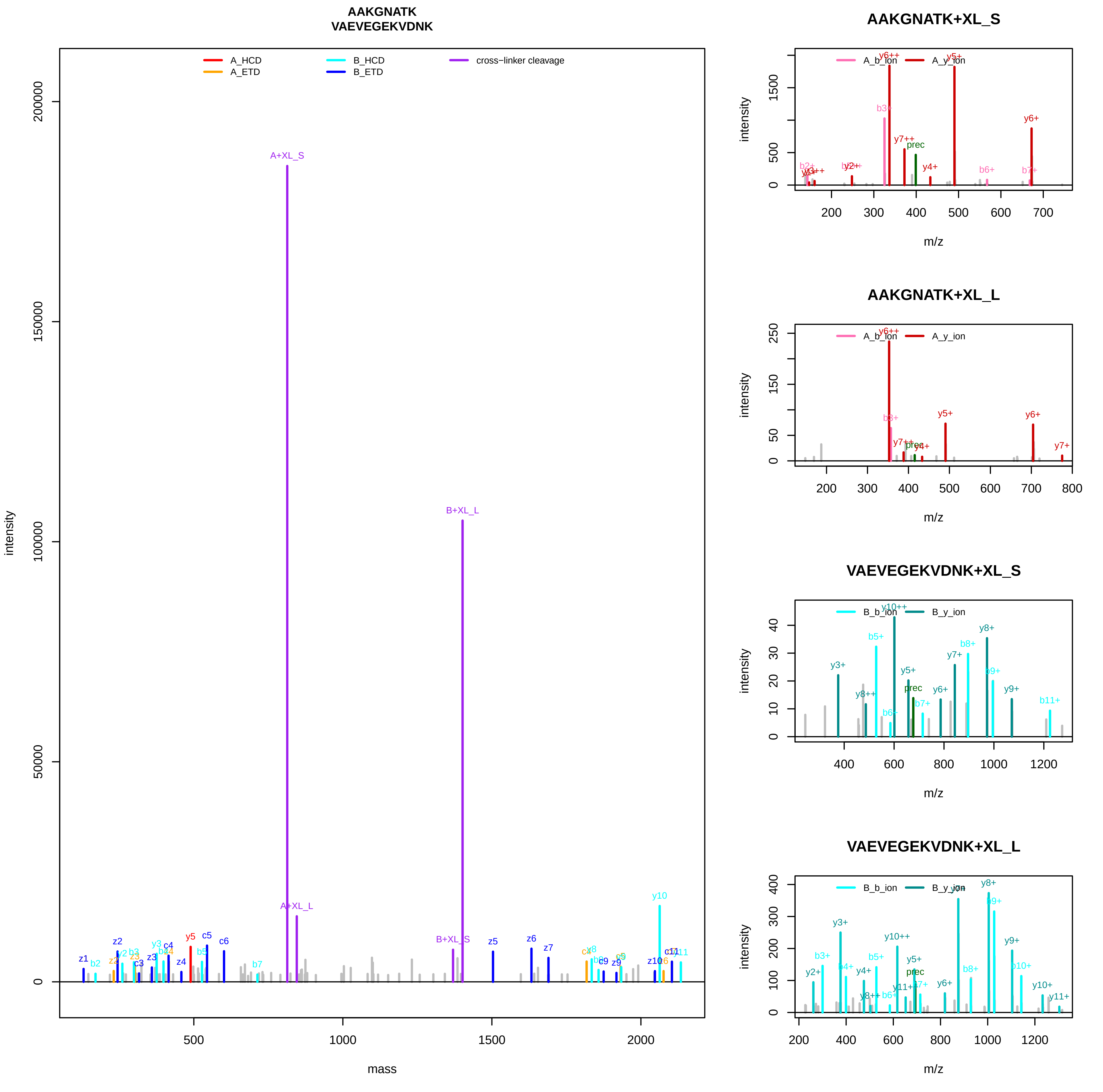
AAKGNATK
VAEVEGEKVDNK
A_HCD
A_ETD
B_HCD
B_ETD
cross−linker cleavage
A+XL_S
B+XL_L
A+XL_L
B+XL_S
y10
intensity
mass
0
50000
100000
150000
200000
500
1000
1500
2000
AAKGNATK+XL_S
A_b_ion
A_y_ion
y6++
y5+
b3+
y7++
prec
y6+
y4+
b6+
b7+
0
500
1500
200
300
400
500
600
700
AAKGNATK+XL_L
y5+
y6+
y7+
0
50
150
250
800
VAEVEGEKVDNK+XL_S
B_b_ion
B_y_ion
y10++
y8+
b8+
b5+
y7+
y3+
y5+
y6+
y9+
b11+
0
10
20
30
40
1000
1200
VAEVEGEKVDNK+XL_L
y9+
y3+
b3+
y2+
b4+
y4+
y10+
100
200
300
400
m/z
intensity
